# Impact of meteorological factors on the incidence of childhood hand, foot, and mouth disease (HFMD) analyzed by DLNMs-based time series approach

**DOI:** 10.1186/s40249-018-0388-5

**Published:** 2018-01-31

**Authors:** Hongchao Qi, Yue Chen, Dongli Xu, Hualin Su, Longwen Zhan, Zhiyin Xu, Ying Huang, Qianshan He, Yi Hu, Henry Lynn, Zhijie Zhang

**Affiliations:** 1 Department of Epidemiology and Biostatistics, School of Public Health, Fudan University, Shanghai, 200032 China; 2 Key Laboratory of Public Health Safety, Ministry of Education, Shanghai, 200032 China; 3 Collaborative Innovation Center of Social Risks Governance in Health, School of Public Health, Fudan University, Shanghai, 200032 China; 4 School of Epidemiology, Pubic Health and Preventive Medicine, Faculty of Medicine, University of Ottawa, 451 Smyth Rd, Ottawa, ON 10610 Canada; 5 Minghang District Center for Disease Control and Prevention, Shanghai, 201101 China; 6 Shanghai Meteorological Service, Shanghai, 201499 China; 7 Shanghai Key Laboratory of Meteorology and Health, Shanghai, 201499 China

**Keywords:** Hand, foot, and mouth disease, Meteorological factor, Distributed lag non-linear model

## Abstract

**Background:**

Hand, foot, and mouth disease (HFMD) has become an emerging infectious disease in China in the last decade. There has been evidence that meteorological factors can influence the HFMD incidence, and understanding the mechanisms can help prevent and control HFMD.

**Methods:**

HFMD incidence data and meteorological data in Minhang District, Shanghai were obtained for the period between 2009 and 2015. Distributed lag non-linear models (DLNMs) were utilized to investigate the impact of meteorological factors on HFMD incidence after adjusting for potential confounders of long time trend, weekdays and holidays.

**Results:**

There was a non-linear relationship between temperature and HFMD incidence, the RR of 5th percentile compared to the median is 0.836 (95% *CI*: 0.671–1.042) and the RR of 95th percentile is 2.225 (95% *CI*: 1.774–2.792), and the effect of temperature varied across age groups. HFMD incidence increased with increasing average relative humidity (%) (RR = 1.009, 95% *CI*: 1.005–1.015) and wind speed (m/s) (RR = 1.197, 95% *CI*: 1.118–1.282), and with decreasing daily rainfall (mm) (RR = 0.992, 95% *CI*: 0.987–0.997) and sunshine hours (h) (RR = 0.966, 95% *CI*: 0.951–0.980).

**Conclusions:**

There were significant relationships between meteorological factors and childhood HFMD incidence in Minhang District, Shanghai. This information can help local health agencies develop strategies for the control and prevention of HFMD under specific climatic conditions.

**Electronic supplementary material:**

The online version of this article (10.1186/s40249-018-0388-5) contains supplementary material, which is available to authorized users.

## Multilingual abstracts

Please see Additional file 1 for translations of the abstract into the six official working languages of the United Nations.

## Background

Hand, foot, and mouth disease (HFMD), a human syndrome caused by highly contagious enteroviruses, was first discovered in New Zealand in 1957 [1]. Since then, HFMD has become an endemic disease in many countries. However, it had been neglected in the Asia-Pacific Region for a long time until its continuous outbreaks in countries and regions such as Malaysia, Taiwan of China, Singapore, Japan, Australia and Republic of Korea at the end of the twentieth century [2–6].

As for China, the first HFMD case was reported in Shanghai in the 1980s [7], but it did not attract public attention until two major outbreaks in 2007 and 2008. In 2007, a HFMD outbreak in Linyi City, Shandong Province led to 1149 cases with three fatalities [8], and another more severe outbreak in Fuyang City, Anhui Province in 2008 resulted in 6049 cases with 20 deaths [9]. A national system to monitor HFMD has been set up since May 2008 [10], and HFMD has become one of the major infectious diseases in the country with an incidence of approximately 1.2 per 1000 person-years and is responsible for a total of 500–900 deaths annually.

As an infectious disease, HFMD can be transmitted through 1) close personal contact, 2) contact with contaminated objects and surfaces, 3) respiratory pathway and 4) fecal-oral pathway (https://www.cdc.gov/hand-foot-mouth/about/transmission.html). Meteorological factors can affect all the transmission channels as well as the survival of enteroviruses in vitro [11, 12]. However, existing studies have shown inconsistent results for the relationships between meteorological factors and HFMD incidence. For instance, a study in Guangzhou showed an approximately linear relationship between temperature and weekly HFMD cases, the weekly HFMD cases increased with weekly average temperature [13], while studies from other areas such as Singapore and Japan [14, 15] revealed non-linear associations in which the relationship varied within the lag range. There are several possible reasons for the discrepancies. First, different modelling schemes (e.g. the statistical model chosen, covariates incorporated in the model) in different studies might lead to different statistical inference results. Second, types of data were different, for example, some studies used daily-based data while others were based on weekly data. Third, region-specific characteristics such as socio-economic factors might result in different behaviors or living environments under similar climatic conditions, which could modify the effects of meteorological factors on HFMD incidence. As a result, more studies conducted in different areas of the Asia-Pacific region are needed to specify the region-specific effects of meteorological factors on HFMD incidence [10].

Distributed lag non-linear models (DLNMs) were used in the current study to quantify the effects of meteorological factors on HFMD incidence in Minhang District, Shanghai due to its flexibility in modelling exposure-response relationship. The objective of the study was to provide more information to health agencies and policy makers for the planning of HFMD control and prevention measures.

## Methods

### Study site

Minhang District is a suburban district of Shanghai with a land area of 372 km^2^ and a population of approximately 2 429 000 (https://en.wikipedia.org/wiki/Minhang_District). It has a mild subtropical climate with four distinctive seasons: spring (March to May), summer (June to August), autumn (September to November) and winter (December to February). The seasonal pattern is suited for exploring the relationship between meteorological factors and HFMD incidence via time series analysis [16].

### Data sources

An individual-based surveillance database including patient’s gender, age and other personal information was obtained from the Minhang District Center for Disease Control and Prevention (CDC) in Shanghai. As over 99% (51 776/52 132) of the patients were younger than 15 years old, the analyses were restricted to those aged less than 15 years. Daily meteorological data from 1st January 2009 to 31st December 2015 were obtained from the Shanghai Meteorological Service including temperature, relative humidity, rainfall, wind speed and sunshine hours (https://en.wikipedia.org/wiki/Sunshine_duration).

### Data preprocessing

Daily HFMD incidence was calculated and matched with meteorological data of the same day. Indicator variables of weekday and holiday on each day during the study period were also created. Holidays included national holidays as well as summer and winter vacations for pupils.

### Statistical analysis

Distributed lag non-linear models (DLNMs) were fitted to detect the relationships between daily count of HFMD cases and meteorological factors [17, 18]. Daily counts of HFMD cases were assumed to have a negative binomial distribution to account for over-dispersion [15]. Daily average temperature was incorporated in the model in the form of a “cross-basis” to account for a possible complex relationship between temperature and HFMD incidence [11, 14]. The cross-basis contains two elements: the natural cubic spline of the non-linear exposure-response relationship and the natural cubic spline of the lag-response relationship. Given that it takes approximately 1 to 7 days from exposure to symptom and diagnosis of HFMD [19], the start lag may be chosen from 1 to 4 days in that a start lag larger than four may underestimate the effect of temperature. According to sensitivity analyses, the start lag was set to four to minimize Akaike Information Criteria (AIC), the result was shown in Additional file 2. Other meteorological variables including relative humidity, wind speed, rainfall and sunshine hours are more likely to have relationships with HFMD incidence in a linear fashion in China [12], and therefore were included in the model as exponential moving averages within the same lag range as temperature’s.

Furthermore, natural cubic splines of calendar time were included in the models to control for long-term trends and seasonality of HFMD incidence. Indicator variables of weekdays and holidays were also incorporated in the models. The model is formulated as follows.

$$ {\displaystyle \begin{array}{l}E\left({Y}_t\right)={\mu}_t\\ {}\log \left({\mu}_t\right)={\beta}_0+ cb\left( Temperature,{df}_1, lag,{df}_2\right)+ EMAs(Meteorological)+ ns\left(t,{df}_3\right)+ factor(weekday)+ factor(holiday)\end{array}} $$

In the formula, *Y*_*t*_ means the daily count of HFMD cases on day *t*, *μ*_*t*_ denotes the expectation of *Y*_*t*_, *β*_0_ stands for the intercept, *cb*() is the cross-basis, *EMA*() means the exponential moving average, *ns*() represents the natural spline, and *factor*() denotes the indicator variables. There are three parameters in the model need to be specified, 2 degrees of freedom (*df*) for the temperature’s cross-basis (*df*_1_ and *df*_2_) and 1 degree of freedom for time’s natural spline (*df*_*3*_). The *df* for exposure-response relationships (*df*_1_) was set to five according similar studies in China [12, 20]. For a total lag of 11 days, a *df* that ranges from 3 to 5 can capture the complexity of the lag structure [12], and *df*_2_ was eventually set to three to minimize AIC. The *df* for time’s natural spline in previous time series studies about chronic health outcomes were usually set to seven [21]. However, HFMD’s time series was more changeable than chronic disease and the degree of freedom could be larger. Therefore, the *df*_3_ for time’s natural spline was set to eight in this study.

The overall exposure-lag-response relationship between temperature and HFMD from lag 4 to lag 14 was specified by comparing to the median of temperature (18.5 °C), and cumulative relative risks of temperature on HFMD were obtained by summing up temperature’s contribution during the lag range (4-14th day). Relative risks on HFMD by temperature at specific lags (4, 7, 10 and 14 days) and by lag at 5th, 25th, 75th and 95th percentile of temperature distribution were also calculated to present the detailed effects of specific temperature at specific lags. Besides, the effects of meteorological variables other than temperature were presented by regression coefficient estimates of their corresponding exponential moving averages.

Stratified analyses were also conducted by gender and age groups (≤ 3 years: usually cared at home; 4–5 years: usually cared in nurseries; 6–14 years: usually attend schools). Children ≤3 years were further divided into two groups (≤ 1 and 2–3 years) due to the difference in daily activities regardless of care type [20].

All the analyses were performed with R 3.3.1 using the *dlnm* and *TTR* package (R Core Team, 2016).

## Results

### Descriptive analysis

A total of 51 776 HFMD cases under 15 years old were recorded in Minhang District, Shanghai from 1st January 2009 to 31st December 2015, of which 31 537 were male and 20 239 were female (male-to-female ratio: 1.56). The overall mean age was 3.05 years, 7.22% (3738/51 776) of the patients aged less than 1 year, 48.95% (25 344/51 776) of those aged 2–3 years, 37.17% (19 244/51 776) of those aged 4–5 years, and 6.66% (3450/51 776) of those aged 6–14 years. Home-cared children accounted for 60.90% (31 533) while nursery school children took up 34.79% (18014), and only 2229 patients (4.31%) were students.

The descriptive statistics for daily HFMD cases and meteorological factors are presented in Table 1. The variance of daily HFMD case count was larger than its average indicating a non-Poisson distribution. Average temperature and average relative humidity were approximately normally distributed while daily rainfall, average wind speed and sunshine hours showed positive skewed distributions.

**Table 1:** Descriptive summary for HFMD cases and meteorological factors in Minhang District, Shanghai, 2009–2015

Variables	Mean	SD	Min	5^th^ Percentile	25^th^ Percentile	Median	75^th^ Percentile	95^th^ Percentile	Max
HFMD incidence									
Daily count	20.3	16.1	0	2	9	16	27	53	106
Daily meteorological variables									
Average temperature (°C)	17.3	8.9	−3.1	2.9	9.3	18.5	24.5	30.4	35.1
Average relative humidity (%)	70.2	12.4	21	48	62	71	79	89	97
Daily rainfall (mm)	3.6	10.9	0	0	0	0	1.4	20.7	195.5
Average wind speed (m/s)	1.8	0.6	0.3	0.9	1.4	1.7	2.2	2.9	5.9
Sunshine hours (h)	5.2	4.1	0	0	0.3	5.7	8.7	11.3	16.3

**Fig. 1:**
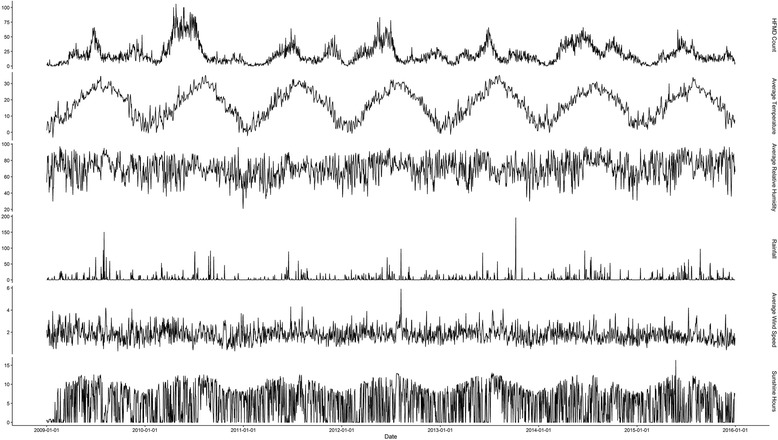
Time-series of hand, foot, and mouth disease (HFMD) and the variations of meteorological variables, 2009–2015

Figure 1 implicitly displays the time series of daily HFMD case count and variations of meteorological factors during the study period. There was a seasonal pattern for daily HFMD case count in Minhang District, Shanghai with 1 or 2 peaks each year. A major peak often occurred in late spring and summer (May–July), and a minor peak might occur in autumn or winter (November–February). The average temperature also had a seasonal pattern, and its peak tended to be earlier than the epidemics of HFMD. There was no explicit seasonal pattern in the time series of other meteorological variables. In addition, the time series of EMAs for meteorological variables are provided in Additional file 3.

### DLNM results

**Fig. 2:**
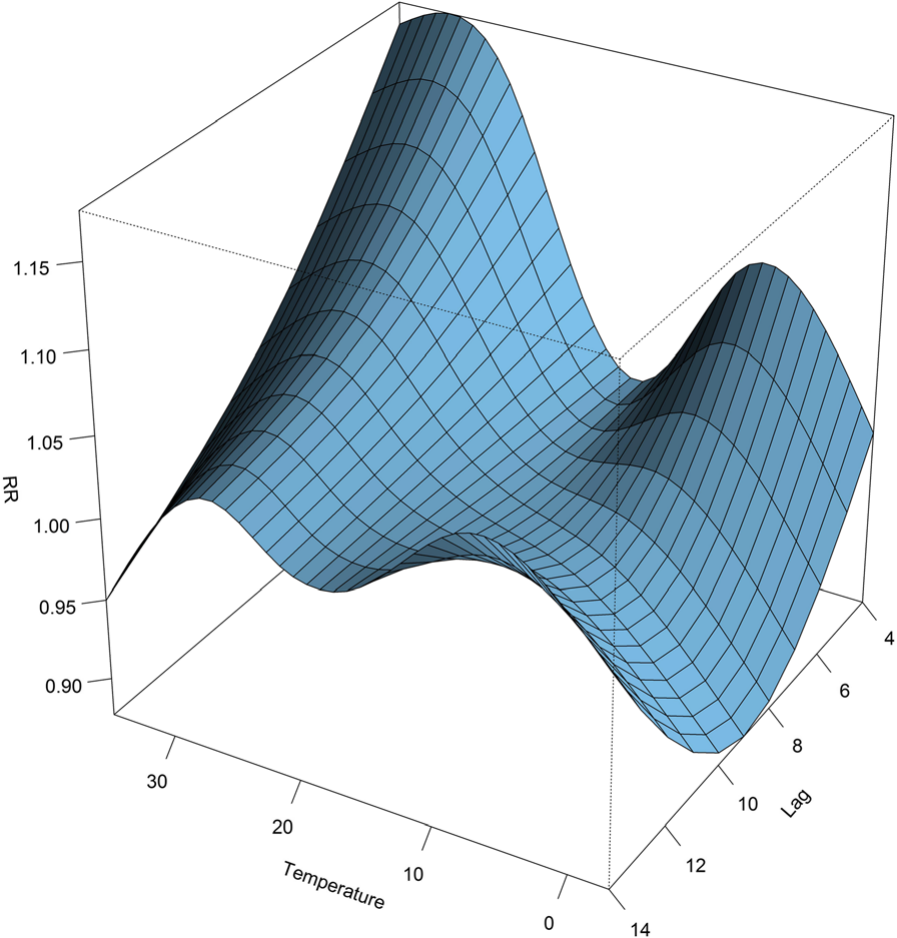
Three-dimensional plot of relative risks (RRs) along temperature and lags, 18.5 °C as reference

Figure 2 is a 3-D plot that depicts the overall exposure-lag-response relationship between temperature and HFMD incidence during the lag range (4th to 14th day). The RRs were calculated with the median of daily average temperature (18.5 °C) as a reference (RR = 1 at 18.5 °C) [22]. The relationship between temperature and HFMD incidence was non-linear, which will be more intuitive after split into slices below.

**Fig. 3:**
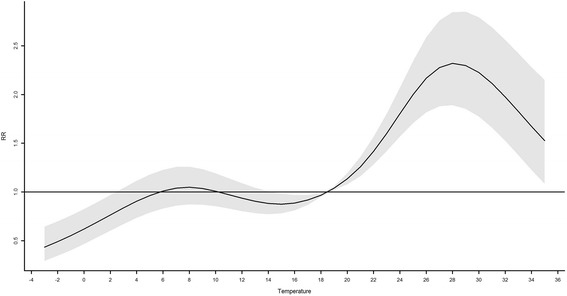
The cumulative relative risks (RRs) of temperature on HFMD, 18.5 °C as reference

Figure 3 shows the cumulative relative risks at different levels of temperature which were calculated by summing up the contribution of temperature during the lag range (from 4th to 14th day). The cumulative relative risk of HFMD incidence reached the peak at the daily average temperature of 28 °C (95% *CI*: 1.78–2.67). Under the temperature of 28 °C, the cumulative relative risk increased with daily average temperature from the minimum to about 2 °C, and then there was little variation in the cumulative relative risk of HFMD from 3 °C to 18.5 °C (5th to 50th percentile). After that, the cumulative relative risk increased with temperature until it reached 28 °C. Above the temperature of 28 °C, the cumulative relative risk decreased with daily average temperature.

**Fig. 4:**
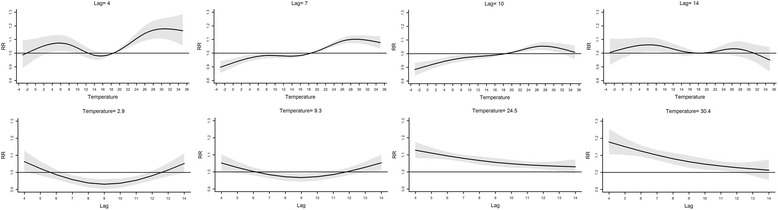
The relative risks (RRs) at specific lags and temperature values, 18.5 °C as reference. Legend of Fig. 4: The upper row of Fig. 4 shows the relative risks (RRs) by temperature at specific lags, and the lower row shows the relative risks (RRs) by lag at 5th, 25th, 75th and 95th percentile of temperature distribution

Figure 4 shows the relative risks for HFMD incidence by daily average temperature at specific lags (4, 7, 10 and 14 days) and by lag at 5th, 25th, 75th and 95th percentile of temperature distribution. At the lag of 4 days, the relative risks were larger than 1 when the temperature was between 2 and 11 °C or higher than 18.5 °C, and otherwise the relative risks were not statistically different from one. At the lag of 7 days, the relative risks were lower than 1 when the temperature was below 18.5 °C, it showed a platform when the temperature was between 8 and 15 °C, and the relative risks increased with the temperature when it was between 18.5 and 28 °C while decreased with the temperature after it exceeded 28 °C. At the lag of 10 days, 28 °C was a turning point, the relative risks increased with temperature before 28 °C and decreased thereafter. At the lag of 14 days, the relative risks were larger than 1 only when the temperature was between 2 and 13 °C. The lower part of Fig. 4 depicts relative risks by lag at 5th, 25th, 75th and 95th percentile of temperature distribution. The relative risks of temperature between 5th and 25th percentile were larger than 1 at the beginning (the lag of 4 days) and end (the lag of 14 days) of the lag range respectively, while the relative risks of temperature between 75th and 95th percentile decreased along lags and became not significantly different from one at the end of the lag range.

**Fig. 5:**
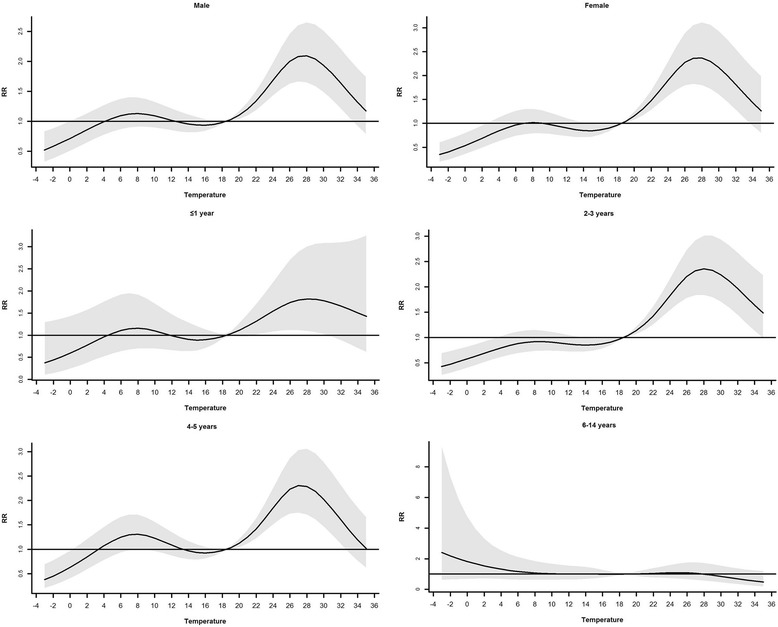
The cumulative relative risks of temperature from different groups, 18.5 °C as reference

Figure 5 depicts the cumulative relative risks associated with temperature stratified by gender and age. The association was similar for males and females except that the maximum of cumulative relative risk was slightly larger for females. For age group ≤1 year, the cumulative relative risks were slightly larger than 1 when the temperature was between 22 and 30.5 °C, and were not significantly different from one otherwise. For age group 2–3 years, the pattern was similar to that of all age groups combined. For age group 4–5 years, there were two peaks for the cumulative relative risk: 3–13 °C and 18.5–32.5 °C. For the age group 6–14 years, the pattern was totally different from the others.

Table 2 shows the relationship between other meteorological factors and HFMD incidence. Average relative humidity and average wind speed were positively, and daily rainfall and sunshine hours were negatively associated with HFMD incidence. When stratified by gender, average relative humidity and average wind speed were positively, and daily rainfall and sunshine hours were negatively associated with HFMD incidence in males; in females, average wind speed was positively, and daily number of sunshine hours was negatively associated with HFMD incidence. For children ≤1 year old, none of the meteorological variable showed statistically significant impact on HFMD incidence; for children aged 2–3 years, average relative humidity and average wind speed had positive effect on HFMD incidence while sunshine hours had negative effect. For children aged 4–5 years, average relative humidity and average wind speed had positive effects on HFMD incidence and for children aged 6–14 years, daily rainfall had negative effect on HFMD incidence. Besides, the estimations of natural spline for time, indicator variables of weekdays and holiday can be found in Additional file 4.

**Table 2:** The coefficient estimates of exponential moving averages of meteorological variables

Variables	Estimate	Standard Error	RR	95% *CI*
Total				
Average relative humidity	0.0098	0.0025	1.009	(1.005, 1.015)
Daily rainfall	−0.0076	0.0026	0.992	(0.987, 0.997)
Average wind speed	0.18	0.035	1.197	(1.118, 1.282)
Sunshine hours	−0.035	0.0078	0.966	(0.951, 0.980)
Male				
Average relative humidity	0.013	0.0029	1.013	(1.007, 1.019)
Daily rainfall	−0.0091	0.0030	0.991	(0.985, 0.997)
Average wind speed	0.16	0.040	1.174	(1.085, 1.269)
Sunshine hours	−0.029	0.0092	0.971	(0.954, 0.989)
Female				
Average relative humidity	0.0044	0.0034	1.004	(0.998, 1.011)
Daily rainfall	−0.0047	0.0035	0.995	(0.989, 1.002)
Average wind speed	0.18	0.047	1.197	(1.092, 1.313)
Sunshine hours	−0.041	0.011	0.960	(0.939, 0.981)
≤ 1 year				
Average relative humidity	0.0096	0.0067	1.010	(0.996, 1.023)
Daily rainfall	−0.010	0.0064	0.990	(0.978, 1.003)
Average wind speed	0.12	0.091	1.127	(0.943, 1.348)
Sunshine hours	−0.029	0.021	0.971	(0.932,1.012)
2–3 years				
Average relative humidity	0.0082	0.0030	1.008	(1.002, 1.014)
Daily rainfall	−0.0059	0.0031	0.994	(0.988, 1.000)
Average wind speed	0.20	0.042	1.221	(1.125, 1.326)
Sunshine hours	−0.045	0.0095	0.956	(0.938, 0.974)
4–5 years				
Average relative humidity	0.014	0.0036	1.014	(1.007, 1.021)
Daily rainfall	−0.0064	0.0037	0.994	(0.986, 1.001)
Average wind speed	0.12	0.050	1.127	(1.022, 1.244)
Sunshine hours	−0.018	0.011	0.982	(0.961, 1.004)
6–14 years				
Average relative humidity	0.0097	0.0073	1.010	(0.995, 1.024)
Daily rainfall	−0.017	0.0074	0.983	(0.969, 0.998)
Average wind speed	0.14	0.10	1.150	(0.946, 1.399)
Sunshine hours	0.014	0.023	1.014	(0.969, 1.061)

## Discussion

This study explored the quantitative relationship between meteorological factors and HFMD incidence in Minhang District, Shanghai between 2009 and 2015. The relationship between temperature and HFMD incidence was nonlinear after adjusting for seasonality, long-term trend and other potential confounders, and it varied across age groups. Average relative humidity and average wind speed showed positive associations, while daily rainfall and sunshine hours showed negative associations with HFMD incidence.

The non-linear relationship between temperature and HFMD incidence has also been found in other studies in China including Hong Kong, Chengdu and Beijing [11, 20, 23], but the pattern in this study is different from those of the three studies mentioned. There are several possible explanations for the observed relationship in this study. Low temperature (lower than 2.2 °C) may not favor the survival of pathogens and personal close contacts, which may hinder the transmission of HFMD [24, 25]. Activities of enteroviruses and frequency of close contacts were similar when the temperature was between 2.2 and 18.5 °C which might lead to a platform of cumulative relative risks. Higher temperature (between 18.5 and 28 °C) may favor the survival of enteroviruses, outdoor activities and close contacts for children, but when the temperature exceeded 28 °C, outdoor activities tended to be less frequent and temperature-sensitive enteroviruses may be less active [12].

The slices of the 3-D plot (see the first row of Fig. 4) illustrated that the relative risks were larger than 1 when the temperature was between 2 and 11 °C at the beginning of the lag range (the lag of 4 days). There might be two reasons for this phenomenon: first, enteroviruses might keep active for about 4 days within this temperature range; second, the indoor ventilation might not as good within this temperature range which may also facilitate the transmission of HFMD [26]. The slices of the 3-D plot (see Fig. 4) also illustrated that the relative risks were larger than 1 when the temperature was between 4 and 10 °C at the end of the lag range (the lag of 14 days), which might be attributed to “harvesting” [27]. The susceptible population can shrink at the early stage of the lag range for temperature higher than 18.5 °C, which may cause the risk of HFMD for high temperature become smaller compared to the risk for low temperature at the final stage of the lag range.

Average relative humidity was positively associated with HFMD incidence, which is consistent with the results from studies conducted in Japan and some areas of China [13, 15, 28, 29]. Humid environment is postulated to favor the attachment of enteroviruses on airborne droplets, which may promote the transmission of HFMD. Enteroviruses also survive longer on the surface of airborne droplets in more humid environment [30]. Daily rainfall had a negative impact on HFMD incidence, which is not in line with the results of another study in China [14, 31]. It was assumed that the pollution of ground water facilitates the transmission of HFMD in that study, while children living in Shanghai are assumed to have a sanitary environment that may protect them from the pollution of ground water. On the contrary, rainfall can flush the airborne droplets which tackles the respiratory pathway of HFMD transmission. Wind speed was positively associated with HFMD incidence, and it is biologically plausible because the transmission is assumed to attribute more to respiratory droplet than the fecal-oral route in developed areas due to better personal hygiene and sanitation facilities [32]. Faster wind speed promotes the respiratory pathway of HFMD, which is in line with a previous study conducted in Hong Kong [33] and Beijing [34]. Sunshine hours were negatively related to HFMD incidence likely because enteroviruses can be inactivated by UV radiation [35]. As a result, periods with increasing average relative humidity and wind speed, and decreasing daily rainfall and sunshine hours are noteworthy.

There are some important findings in the stratified analyses. The cumulative risk of 28 °C was slightly larger for females than males but there were more male cases which was also found in Beijing [20]. This result indicated that there were other risk factors for males. Boys are usually more physically active than girls, which may increase the probability of HFMD transmission via close contact. Furthermore, there may be gender-related difference in HFMD susceptibility, which needs further exploration. The phenomenon indicates that maybe more attentions should be paid to boys due to their susceptibility to the disease. Children ≤1 year were less influenced by meteorological factors, which might result from the protection of maternal antibodies [36]. Besides, these children are usually cared at home, this certain pattern of care may protect them from getting infected via close personal contact. For children aged between 2 and 3 years, the pattern of the temperature and HFMD association was similar to that of all age groups combined. For children aged between 4 and 5 years, the pattern of the temperature and HFMD association was in a “M” shape, i.e., the risk was relatively high for the temperature between 3 and 13 °C. The potential explanation might be that the indoor ventilation in nurseries (for most children at the age range) is inadequate within this temperature range which promotes the transmission of HFMD via respiratory pathway [26]. For children aged between 6 and 14 years, the association pattern was very different from those of other age groups, the risk of HFMD was highest within lowest temperature range though the risk was not statistically significant due to the wide confidence interval. The possible explanation is that the small sample size for this age group could be strongly influenced by outliers (e.g., four cases were reported on a day with a daily average temperature − 0.8 °C while the average daily incidence for 18.5 °C is 0.67). Besides, the excessively wide confidence interval could also be attributed to the sparseness of cases in this age group.

Due to the discrepancies of patterns of HFMD risk across different age groups, specific interventions should be applied to different age groups by local caregivers and health agencies. For instance, boys require more attentions during the prevention because of their vulnerability to the disease, and babysitters in the nurseries should take prophylactic measures, like sterilization and ventilation within 3 to 13 °C. Besides, disease prevention staffs should reinforce health education and supervision of nurseries within this temperature range; and pediatrician should be alerted and prepared for the epidemics of HFMD.

One limitation of the study is that serotypes of enteroviruses were not specified in the data. In China, the epidemics of enteroviruses other than EV71 and CA16 have been rising recently [37, 38]. Different genotypes of enteroviruses may result in different health outcomes, on which meteorological factors may have different impacts. Information about the serotypes of HFMD pathogens can help clarify this issue. However, this study has some important strength including regulated data collection and management, long study period and large study population.

## Conclusions

This study has demonstrated that there was a non-linear relationship between daily average temperature and HFMD incidence in Minhang District, Shanghai. The most important finding in this study was that the effects of daily average temperature on HFMD incidence varied across different age groups. In addition to special attentions to the prevention and control of HFMD during relatively warm days (higher than 18.5 °C), the result has revealed that it may also be necessary to apply special interventions to children cared in nurseries when the temperature ranges from 3 to 13 °C.

## Additional files

Additional file 1: Multilingual abstracts in the six official working languages of the United Nations. (PDF 757 kb)

Additional file 2: The comparison of model fit statistics among different lag ranges. (PDF 97 kb)

Additional file 3: The time series of EMAs for four meteorological variables. (PDF 382 kb)

Additional file 4: The estimation of natural spline for time, indicator variables of weekdays and holiday. (PDF 147 kb)

## Abbreviations

AIC: Akaike Information Criteria
CDC: Center for disease control and prevention
DLNM: Distributed lag non-linear model
EMA: Exponential moving average
HFMD: Hand, foot, and mouth disease
RR: Relative risk

## References

[1] Xu M, Su L, Cao L, Zhong H, Dong N, Xu J (2013). Enterovirus genotypes causing hand foot and mouth disease in shanghai, China: a molecular epidemiological analysis. BMC Infect Dis.

[2] Fujimoto T, Chikahira M, Yoshida S, Ebira H, Hasegawa A, Totsuka A (2002). Outbreak of central nervous system disease associated with hand, foot, and mouth disease in Japan during the summer of 2000: detection and molecular epidemiology of enterovirus 71. Microbiol Immunol.

[3] Chan KP, Goh KT, Chong CY, Teo ES, Lau G, Ling AE (2003). Epidemic hand, foot and mouth disease caused by human enterovirus 71, Singapore. Emerg Infect Dis.

[4] Jee YM, Cheon DS, Kim K, Cho JH, Chung YS, Lee J (2003). Genetic analysis of the VP1 region of human enterovirus 71 strains isolated in Korea during 2000. Arch Virol.

[5] Ho M, Chen ER, Hsu KH, Twu SJ, Chen KT, Tsai SF (1999). An epidemic of enterovirus 71 infection in Taiwan. Taiwan Enterovirus epidemic working group. N Engl J Med.

[6] Chan LG, Parashar UD, Lye MS, Ong FG, Zaki SR, Alexander JP (2000). Deaths of children during an outbreak of hand, foot, and mouth disease in sarawak, malaysia: clinical and pathological characteristics of the disease. For the outbreak study group. Clin Infect Dis.

[7] Mao QY, Wang YP, Bian LL, Xu M, Liang ZL (2016). EV71 vaccine, a new tool to control outbreaks of hand, foot and mouth disease (HFMD). Expert Rev Vaccines.

[8] Zhang Y, Tan XJ, Wang HY, Yan DM, Zhu SL, Wang DY (2009). An outbreak of hand, foot, and mouth disease associated with subgenotype C4 of human enterovirus 71 in Shandong, China. J Clin Virol.

[9] Zhang Y, Zhu Z, Yang W, Ren J, Tan X, Wang Y (2010). An emerging recombinant human enterovirus 71 responsible for the 2008 outbreak of hand foot and mouth disease in Fuyang city of China. Virol J.

[10] Xing W, Liao Q, Viboud C, Zhang J, Sun J, Wu JT (2014). Hand, foot, and mouth disease in China, 2008-12: an epidemiological study. Lancet Infect Dis.

[11] Wang P, Goggins WB, Chan EY (2016). Hand, foot and mouth disease in Hong Kong: a time-series analysis on its relationship with weather. PLoS One.

[12] Xiao X, Gasparrini A, Huang J, Liao Q, Liu F, Yin F (2017). The exposure-response relationship between temperature and childhood hand, foot and mouth disease: a multicity study from mainland China. Environ Int.

[13] Huang Y, Deng T, Yu S, Gu J, Huang C, Xiao G (2013). Effect of meteorological variables on the incidence of hand, foot, and mouth disease in children: a time-series analysis in Guangzhou, China. BMC Infect Dis.

[14] Hii YL, Rocklov J, Ng N (2011). Short term effects of weather on hand, foot and mouth disease. PLoS One.

[15] Onozuka D, Hashizume M (2011). The influence of temperature and humidity on the incidence of hand, foot, and mouth disease in Japan. Sci Total Environ.

[16] Jiang FC, Yang F, Chen L, Jia J, Han YL, Hao B (2016). Meteorological factors affect the hand, foot, and mouth disease epidemic in Qingdao, China, 2007–2014. Epidemiol Infect.

[17] Gasparrini A, Armstrong B, Kenward MG (2010). Distributed lag non-linear models. Stat Med.

[18] Gasparrini A (2014). Modeling exposure-lag-response associations with distributed lag non-linear models. Stat Med.

[19] Goh KT, Doraisingham S, Tan JL, Lim GN, Chew SE (1982). An outbreak of hand, foot, and mouth disease in Singapore. Bull World Health Organ.

[20] Xu MM, Yu WW, Tong SL, Jia L, Liang FC, Pan XC. Non-linear association between exposure to ambient temperature and children's hand-foot-and-mouth disease in Beijing, China. PLoS One. 2015;10(5):e0126171 10.1371/journal.pone.0126171 PMC4444089 26010147

[21] Peng RD, Dominici F, Louis TA (2006). Model choice in time series studies of air pollution and mortality. J Royal Stat Soc Series A-Stat Soc.

[22] Zhu L, Wang X, Guo Y, Xu J, Xue F, Liu Y (2016). Assessment of temperature effect on childhood hand, foot and mouth disease incidence (0-5years) and associated effect modifiers: a 17 cities study in Shandong Province, China, 2007-2012. Sci Total Environ.

[23] Yin F, Zhang T, Liu L, Lv Q, Li XS (2016). The association between ambient temperature and childhood hand, foot, and mouth disease in Chengdu, China: a distributed lag non-linear analysis. Sci Rep.

[24] Rajtar B, Majek M, Polanski L, Polz-Dacewicz M (2008). Enteroviruses in water environment - a potential threat to public health. Ann Agr Env Med.

[25] Suminski RR, Poston WC, Market P, Hyder M, Sara PA (2008). Meteorological conditions are associated with physical activities performed in open-air settings. Int J Biometeorol.

[26] Hobday RA, Dancer SJ (2013). Roles of sunlight and natural ventilation for controlling infection: historical and current perspectives. J Hosp Infect.

[27] Rabl A (2005). Air pollution mortality: harvesting and loss of life expectancy. J Toxicol Environ Health A.

[28] Lin H, Zou H, Wang Q, Liu C, Lang L, Hou X (2013). Short-term effect of el Nino-southern oscillation on pediatric hand, foot and mouth disease in Shenzhen, China. PLoS One.

[29] Wei J, Hansen A, Liu Q, Sun Y, Weinstein P, Bi P (2015). The effect of meteorological variables on the transmission of hand, foot and mouth disease in four major cities of Shanxi Province, China: a time series data analysis (2009-2013). PLoS Negl Trop Dis.

[30] Kramer A, Schwebke I, Kampf G (2006). How long do nosocomial pathogens persist on inanimate surfaces? A systematic review. BMC Infect Dis.

[31] Wang Y, Feng Z, Yang Y, Self S, Gao Y, Longini IM (2011). Hand, foot, and mouth disease in China: patterns of spread and transmissibility. Epidemiology.

[32] Wong SS, Yip CC, Lau SK, Yuen KY (2010). Human enterovirus 71 and hand, foot and mouth disease. Epidemiol Infect.

[33] Ma E, Lam T, Wong C, Chuang SK (2010). Is hand, foot and mouth disease associated with meteorological parameters?. Epidemiol Infect.

[34] Wu X, Sun Y, Lin C, Jia L, Wu Q, Li X (2014). A case-control study to identify environmental risk factors for hand, foot, and mouth disease outbreaks in Beijing. Japan J Infect Dis.

[35] Bertrand I, Schijven JF, Sanchez G, Wyn-Jones P, Ottoson J, Morin T (2012). The impact of temperature on the inactivation of enteric viruses in food and water: a review. J Appl Microbiol.

[36] Ooi EE, Phoon MC, Ishak B, Chan SH (2002). Seroepidemiology of human enterovirus 71, Singapore. Emerg Infect Dis.

[37] He YQ, Chen L, Xu WB, Yang H, Wang HZ, Zong WP (2013). Emergence, circulation, and spatiotemporal phylogenetic analysis of coxsackievirus a6-and coxsackievirus a10-associated hand, foot, and mouth disease infections from 2008 to 2012 in Shenzhen, China. J Clin Microbiol.

[38] Xu M, Su L, Cao L, Zhong H, Dong N, Dong Z (2015). Genotypes of the enterovirus causing hand foot and mouth disease in shanghai, China, 2012-2013. PLoS One.

