# Supplementary material for: Impact of meteorological factors on the incidence of childhood hand, foot, and mouth disease (HFMD) analyzed by DLNMs-based time series approach
Source: Infect Dis Poverty. 2018 Jan 31;7:7. doi: 10.1186/s40249-018-0388-5 (PMC5796399; doi:10.1186/s40249-018-0388-5)
Supplement: Supplementary file 1 — Multilingual abstracts in the six official working languages of the United Nations. (PDF 757 kb) [file 40249_2018_388_MOESM1_ESM.pdf]

Translation of the abstract into the six official working languages of the United Nations

تأثير عوامل الأرصاد الجوية على معدل الإصابة بأمراض اليد والقدم والفم في مرحلة الطفولة التي تم تحليلها بواسطة المتواليات الزمنية القائمة على نماذج التأخر غير الخطية الموزعة

هونج تشاو تشي، يو تشن، دونجلي شو، هوالين سو، لونجون زان، زيين شو، يينج هوانج، كيانشان هو، يي هو جين تاو، هنري لين، زيجي تشانج

ملخص

خلفية: أصبح مرض اليد والقدم والفم مرضا معديا ناشئا في الصين في العقد الماضي. وقد كان هناك دليل على أن عوامل الأرصاد الجوية يمكن أن تؤثر على الإصابة بمرض اليد والقدم والفم، وعلى فهم آليات يمكن أن تساعد في السيطرة والوقاية من مرض اليد والقدم والفم.

الأساليب: تم الحصول على بيانات الإصابة بالعدوى بمرض اليد والقدم والفم في منطقة مينهانج في شانغهاي للفترة بين عامي 2009 و2015. واستخدمت نماذج التأخر غير الخطية الموزعة لدراسة تأثير عوامل الأرصاد الجوية على الإصابة بمرض اليد والقدم والفم بعد ضبط العوامل المحيرة المحتملة من للاتجاهات الزمنية طويلة الأمد، وأيام الأسبوع والأعياد.

النتائج: كانت هناك علاقة غير خطية بين درجة الحرارة ومرض اليد والقدم والفم، والمخاطر النسبية من المرتبة المئوية الخامسة مقارنة بالمتوسط وهو 0.836 (95٪ حد الثقة: 1.042-0.671) والمخاطر النسبية من المرتبة المئوية 95 وهو 2.225 (95٪ حد الثقة: 2.792-1.774)، وتفاوت تأثير درجة الحرارة عبر الفئات العمرية. زادت نسبة الإصابة بمرض اليد والقدم والفم بزيادة متوسط الرطوبة النسبية (٪) (المخاطر النسبية = 1.009، 95٪ حد الثقة: 1.015-1.005) وسرعة الرياح (المخاطر النسبية = 1.197، 95٪ حد الثقة: 1.282-1.118)، مع انخفاض معدل هطول الأمطار اليومي (مم) (المخاطر النسبية = 0.992، 95٪ حد الثقة: 0.997-0.987) وساعات سطوع الشمس (س) (المخاطر النسبية = 0.966، 95٪ حد الثقة: 0.980-0.951).

الاستنتاجات: كانت هناك علاقات كبيرة بين عوامل الأرصاد الجوية والإصابة بمرض اليد والقدم والفم في الأطفال، في منطقة مينهانج، شانغهاي. ويمكن لهذه المعلومات أن تساعد المؤسسات الصحية المحلية على وضع استراتيجيات لمكافحة والوقاية من مرض اليد والقدم والفم في ظل ظروف مناخية محددة.

Translated from English version into Arabic by Mahmoud Sami, through

TRANSLATORS
WITHOUT BORDERS

利用分布滞后非线性模型（DLNM）分析气象因素对儿童手足口病发病率的影响

Hongchao Qi, Yue Chen, Dongli Xu, Hualin Su, Longwen Zhan, Zhiyin Xu, Ying Huang, Qianshan He, Yi Hu, Henry Lynn, Zhijie Zhang

摘要

引言：手足口病是中国近十年来的新发传染病。现有的研究证据表明气象因素会影响手足口病的发病，因此理解气象因素对手足口病的发病的影响机制可以辅助手足口病的防控工作。

方法：本研究利用分布滞后非线性模型（DLNM），在控制了长期时间趋势，工作日和假期的混杂的情况下，分析上海市闵行区2009~2015年期间的手足口病发病数据和气象数据以量化气象因素对手足口病发病的影响。

结果：本研究表明气温和手足口病存在非线性关系，气温的第5百分位数相对于气温的中位数的RR值为0.836（95% *CI*: 0.671-1.042）而气温的第95百分位数相对于中位数的RR值为2.225（95% *CI*: 1.774-2.792），且温度效应在不同的年龄组中不一致。手足口病发病数随着平均相对湿度（%）（RR=1.009, 95% *CI*: 1.005-1.015）和风速（m/s）（RR=1.197, 95% *CI*: 1.118-1.282）的增加而增加，而与每日平均降雨量（mm）（RR=0.992, 95% *CI*: 0.987-0.997）和日照时间（h）（RR=0.966, 95% *CI*: 0.951-0.980）呈负相关。
结论：研究表明在上海市闵行区气象因素和儿童手足口病发病之间的关系有显著的统计学意义。该研究提供的信息可以帮助当地的卫生机构在特定的气象条件下制定手足口病的防控策略。

Translated from English version into Chinese by Hongchao Qi

Impact des facteurs météorologiques sur l'incidence du pied, main, bouche chez l'enfant (HFMD) analysé par l'approche chronologique basée sur le DNLM

Hongchao Qi, Yue Chen, Dongli Xu, Hualin Su, Longwen Zhan, Zhiyin Xu, Ying Huang, Qianshan He, Yi Hu, Henry Lynn, Zhijie Zhang

Résumé
Contexte: Le pied main bouche (HFMD) est devenu une maladie émergente en Chine ces dix dernières années. Des preuves existent que les facteurs météorologiques influencent l'incidence du HFMD, et en comprendre les mécanismes peut aider à contrôler et prévenir le HFMD.
Méthodes: Les données d'incidence du HFMD et les données médicales dans le district Minhang, Shangai, ont été obtenues pour la période allant de 2009 à 2015. Le décalage Distribué des modèles non-linéaires (DLNMs) a été utilisé pour examiner l'impact de facteurs météorologiques sur l'incidence du HFMD après le réglage pour les facteurs de confusion potentiels de tendance de longue période de temps, des jours ouvrables et des jours fériés.
Résultats: Une relation non linéaire a été mise en évidence entre la température et l'incidence du HFMD, le RR du 5eme percentile comparé au médian est 0.836 (95% *CI* :0.671-1.042) et le RR de 95eme percentile est 2.225 (95% *CI*:1.774-2.792), et l'effet de la température varie selon les groupes d'âge. L'incidence du HFMD a augmentée avec la moyenne croissante de l'humidité relative (%) (RR=1.009 95% *CI*: 1.005-1.015) et vitesse du vent (m/s) (RR=1.197, 95% *CI*: 1.118-1.282), et avec des précipitations quotidiennes diminuant (mm) (RR=0.992, 95% *CI*: 0.987-0.997) et les heures d'ensoleillement (h) (RR=0.966, 95% *CI*: 0.951-0.980).
Conclusions: Des liens significatifs entre les facteurs météorologiques et l'incidence du HFMD infantile dans le district de Minhang, Shangai. Cette information peut aider les agences locales à développer des stratégies pour le contrôle et la prévention du HFMD dans des conditions climatiques particulières.

Translated from English version into French by Emilie Rigault Fourcadier, through

TRANSLATORS
WITHOUT BORDERS

Влияние метеорологических факторов на частоту развития энтеровирусного везикулярного стоматита (HFMD) у детей, выявленного в результате анализа данных, полученных при использовании подхода, основанного на нелинейных моделях с распределенной задержкой

Хонгчао Кви/Hongchao Qi, Ю Чен/Yue Chen, Донги Ксу/Dongli Xu, Хуалин Су/Hualin Su, Лонгвен Жан/Longwen Zhan, Жийин Ксу/Zhiyin Xu, Инг Хуанг/Ying Huang, Квианшан Хе/Qianshan He, И Ху/Yi Hu, Генри Линн/Henry Lynn, Жийе Жанг/Zhijie Zhang

Краткое изложение

Исходная информация: За последние десятилетия энтеровирусный везикулярный стоматит (HFMD) стал возникающим инфекционным заболеванием в Китае. Были выявлены признаки того, что метеорологические факторы могут оказывать влияние на частоту развития энтеровирусного везикулярного стоматита (HFMD). Также выяснили, что понимание механизмов развития заболевания может способствовать контролю и профилактике энтеровирусного везикулярного стоматита.

Методы: Данные о частоте развития энтеровирусного везикулярного стоматита и метеорологические данные из района Миньхан, Шанхай, были получены в период с 2009 по 2015 гг. Для изучения влияния метеорологических факторов на частоту развития энтеровирусного везикулярного стоматита использовали нелинейные модели с распределенной задержкой (DLNMs) с учетом потенциальных факторов, искажающих результаты при продолжительных исследованиях, выходных и праздничных дней.

Результаты: Наблюдалась нелинейная связь между температурой и частотой развития энтеровирусного везикулярного стоматита, диапазон нормальных значений (RR) от 5-й процентили составил 0,836 (95% *доверительный интервал*: 0,671-1,042) по сравнению со срединным значением, а диапазон нормальных значений от 95-й процентили составил 2,225 (95% *доверительный интервал*: 1,774-2,792), действие температуры изменялось в зависимости от возрастной группы. Частота развития энтеровирусного везикулярного стоматита повышалась при повышении средней относительной влажности (%) (RR=1,009, 95% *доверительный интервал*: 1,005-1,015) и скорости ветра (м/с) (RR=1,197, 95% *доверительный интервал*: 1,118-1,282), и при уменьшении суточного количества осадков (мм) (RR=0,992, 95% *доверительный интервал*: 0,987-0,997) и длительности светового дня (ч) (RR=0,966, 95% *доверительный интервал*: 0,951-0,980).

Выводы: Наблюдалась значимая связь между метеорологическими факторами и частотой развития энтеровирусного везикулярного стоматита у детей в районе Миньхан, Шанхай. Эта информация может помочь местным организациям здравоохранения разработать стратегии, которые позволят осуществлять контроль и профилактику развития энтеровирусного везикулярного стоматита в специфических климатических условиях.

Translated from English version into Russian by Helga_Sh, through

El impacto de factores meteorológicos en la incidencia de la enfermedad de boca-mano-pie (o HFMD por sus siglas en inglés) en la infancia, analizado según un enfoque basado en modelos no lineales de retardos distribuidos en series temporales.

Hongchao Qi, Yue Chen, Dongli Xu, Hualin Su, Longwen Zhan, Zhiyin Xu, Ying Huang, Qianshan He, Yi Hu, Henry Lynn, Zhijie Zhang

Resumen

Antecedentes: la enfermedad de boca-mano-pie (HFMD) se ha convertido en una enfermedad infecciosa emergente en China en la última década. Se han hallado pruebas de que los factores meteorológicos pueden tener una influencia en la incidencia de HFMD, por lo que entender esos mecanismos puede ayudar a controlar y prevenir la HFDM.

Métodos: La información sobre la incidencia de HFMD y la información meteorológica en el Distrito Minhang de Shanghái se recabaron para el período entre 2009 y 2015. Se utilizaron modelos no lineales de retardos distribuidos para investigar el impacto de factores meteorológicos en la incidencia de HFMD teniendo en cuenta potenciales factores de confusión de tendencia secular, días entre semana y vacaciones.

Resultados: Se constató una relación no lineal entre la temperatura y la incidencia de HFMD, el riesgo relativo (RR) del percentil 5 comparado con la media es 0,836 (95% Intervalo de Confianza (IC): 0,671-1,042) y el RR del percentil 95 es de 2,225 (95% *IC:* 1,774-2,792). El efecto de la temperatura fue variable en los diferentes grupos de edad. La incidencia de HFMD fue mayor al aumentar la humedad relativa media (%) (RR=1,009; 95% *IC*: 1,005-1,015) y la velocidad del viento (m/s) (RR=1,197; 95% *IC:* 1,118-1,282), y al disminuir las precipitaciones diarias (mm) (RR=0,992; 95% *IC*: 0,987-0,997) y las horas de sol (h) (RR=0,966; 95% *IC*: 0,951-0,980).

Conclusiones: Se constataron relaciones significativas entre los factores meteorológicos y la incidencia de la HFMD infantil en el Distrito de Minhang, en Shanghái. Esta información puede ayudar a las agencias de salud locales a desarrollar estrategias para controlar y prevenir la enfermedad de boca-mano-pie bajo unas condiciones climáticas específicas.

Translated from English version into Spanish by Yaiza Jurado, through
