# Supplementary material for: Impact of meteorological factors on the incidence of childhood hand, foot, and mouth disease (HFMD) analyzed by DLNMs-based time series approach
Source: Infect Dis Poverty. 2018 Jan 31;7:7. doi: 10.1186/s40249-018-0388-5 (PMC5796399; doi:10.1186/s40249-018-0388-5)
Supplement: Supplementary file 2 — The comparison of model fit statistics among different lag ranges. (PDF 97 kb) [file 40249_2018_388_MOESM2_ESM.pdf]

Table 1. The comparison of model fit statistics among different lag ranges.

Candidates of lag ranges	AIC
0-14 days	163901.5
1-14 days	156525.7
2-14 days	152358.9
3-14 days	173325.4
4-14 days	150978.8
