# Supplementary figures and images for: Impact of meteorological factors on the incidence of childhood hand, foot, and mouth disease (HFMD) analyzed by DLNMs-based time series approach
Source: Infect Dis Poverty. 2018 Jan 31;7:7. doi: 10.1186/s40249-018-0388-5 (PMC5796399; doi:10.1186/s40249-018-0388-5)

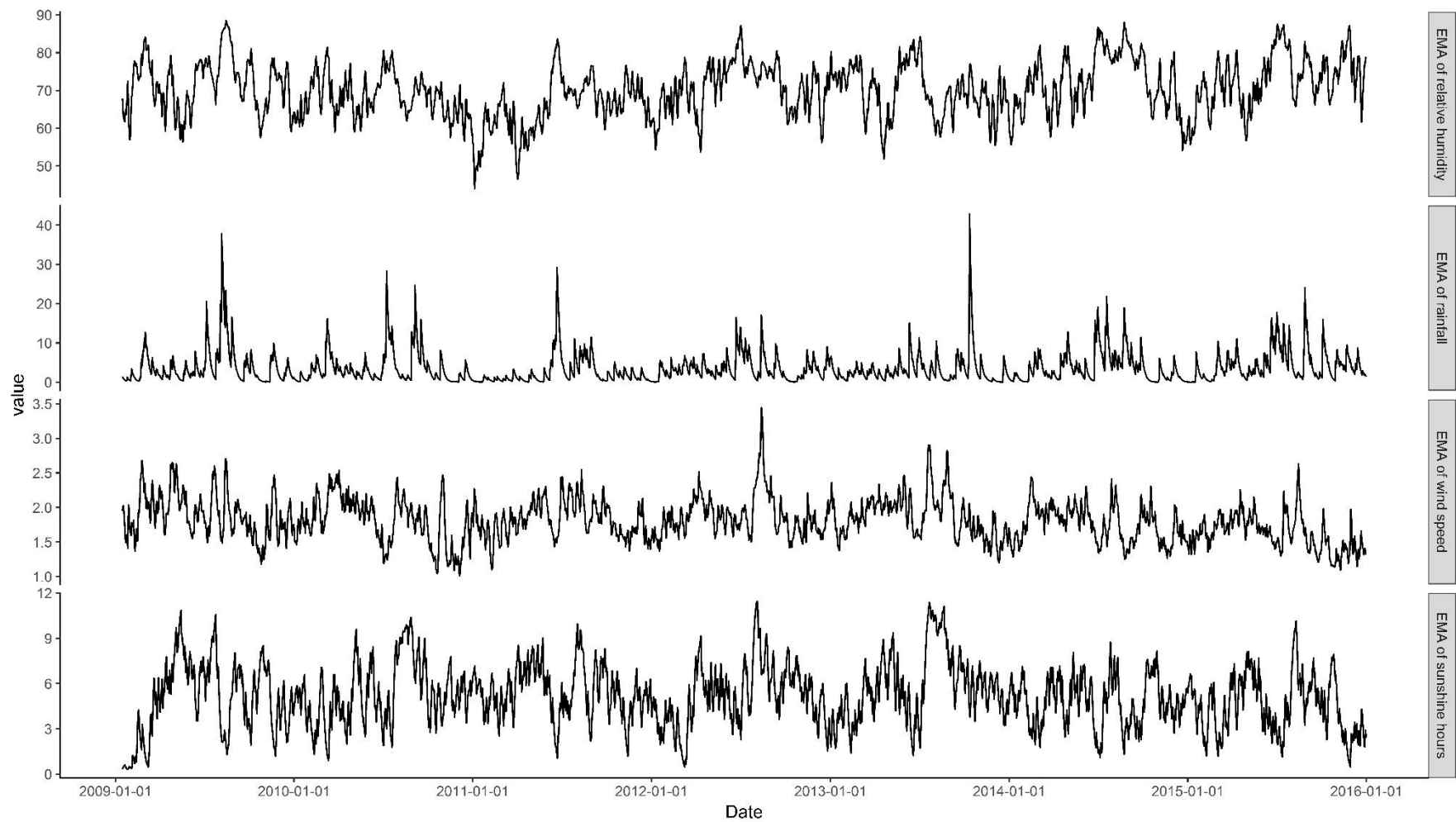

Figure 1. The time series of EMAs for four meteorological variables.

Supplement: Supplementary file 3 — The time series of EMAs for four meteorological variables. (PDF 382 kb) [file 40249_2018_388_MOESM3_ESM.pdf]
