# Supplementary material for: Impact of meteorological factors on the incidence of childhood hand, foot, and mouth disease (HFMD) analyzed by DLNMs-based time series approach
Source: Infect Dis Poverty. 2018 Jan 31;7:7. doi: 10.1186/s40249-018-0388-5 (PMC5796399; doi:10.1186/s40249-018-0388-5)
Supplement: Supplementary file 4 — The estimation of natural spline for time, indicator variables of weekdays and holiday. (PDF 147 kb) [file 40249_2018_388_MOESM4_ESM.pdf]

Table 1. The estimation of natural spline for time, indicator variables of weekdays and holiday.

Variables		Estimate	Standard error	RR	95% CI
Natural spline of time	1	3.6711	0.2524	39.2951	(23.9602,64.4445)
Natural spline of time	2	2.19	0.3573	8.9352	(4.4357,17.9989)
Natural spline of time	3	3.3019	0.3198	27.1642	(14.5137,50.8412)
Natural spline of time	4	0.8439	0.3403	2.3254	(1.1935,4.5308)
Natural spline of time	5	2.6969	0.3276	14.8337	(7.8053,28.1908)
Natural spline of time	6	3.1376	0.3105	23.0485	(12.5412,42.359)
Natural spline of time	7	2.8963	0.2955	18.107	(10.1464,32.3133)
Natural spline of time	8	1.5684	0.304	4.799	(2.6447,8.708)
Natural spline of time	9	4.7107	0.3027	111.1299	(61.3999,201.1382)
Natural spline of time	10	3.724	0.3244	41.4298	(21.937,78.2434)
Natural spline of time	11	3.6345	0.3246	37.8829	(20.0511,71.5729)
Natural spline of time	12	1.4621	0.3541	4.315	(2.1556,8.6377)
Natural spline of time	13	1.6811	0.3246	5.3715	(2.8431,10.1484)
Natural spline of time	14	2.959	0.3217	19.2787	(10.2622,36.2171)
Natural spline of time	15	2.146	0.3044	8.5506	(4.7085,15.5277)
Natural spline of time	16	-0.312	0.3084	0.732	(0.3999,1.3397)
Natural spline of time	17	3.3165	0.309	27.5637	(15.0422,50.5084)
Natural spline of time	18	2.7137	0.3257	15.085	(7.9672,28.5619)
Natural spline of time	19	3.0429	0.3244	20.966	(11.1015,39.5958)
Natural spline of time	20	1.3308	0.3414	3.7841	(1.938,7.3886)
Natural spline of time	21	1.502	0.3186	4.4907	(2.405,8.3851)
Natural spline of time	22	4.5499	0.3074	94.6229	(51.8003,172.8465)

Natural spline of time	23	0.9473	0.3016	2.5787	(1.4278,4.6573)
Natural spline of time	24	2.6209	0.3018	13.7481	(7.6093,24.8393)
Natural spline of time	25	3.4049	0.3074	30.1113	(16.4841,55.0039)
Natural spline of time	26	3.4164	0.3255	30.4596	(16.0936,57.6494)
Natural spline of time	27	3.1406	0.3262	23.1177	(12.1977,43.8139)
Natural spline of time	28	0.3276	0.3442	1.3876	(0.7068,2.7244)
Natural spline of time	29	2.5709	0.3322	13.0776	(6.8195,25.0785)
Natural spline of time	30	2.5776	0.314	13.1655	(7.1147,24.3624)
Natural spline of time	31	3.5479	0.3009	34.7403	(19.262,62.6563)
Natural spline of time	32	0.5238	0.3049	1.6884	(0.9289,3.0692)
Natural spline of time	33	4.2314	0.3065	68.8135	(37.7377,125.4792)
Natural spline of time	34	1.5751	0.3199	4.8312	(2.5808,9.044)
Natural spline of time	35	4.0173	0.3291	55.5509	(29.1444,105.8831)
Natural spline of time	36	1.0441	0.3464	2.8408	(1.4407,5.6016)
Natural spline of time	37	2.6855	0.3389	14.6655	(7.5478,28.4954)
Natural spline of time	38	2.8522	0.3086	17.3259	(9.4626,31.7234)
Natural spline of time	39	2.2259	0.3039	9.2618	(5.1052,16.8028)
Natural spline of time	40	0.4106	0.3035	1.5077	(0.8317,2.7332)
Natural spline of time	41	4.7541	0.3055	116.0592	(63.7724,211.2157)
Natural spline of time	42	2.8222	0.312	16.8138	(9.1219,30.9917)
Natural spline of time	43	3.6523	0.3308	38.5633	(20.1647,73.749)
Natural spline of time	44	1.4112	0.3317	4.1009	(2.1406,7.8564)
Natural spline of time	45	3.512	0.3341	33.5152	(17.4121,64.511)
Natural spline of time	46	2.5164	0.3071	12.3839	(6.7834,22.6083)

Natural spline of time	47	3.2218	0.3046	25.0732	(13.8016,45.5502)
Natural spline of time	48	-0.8065	0.3064	0.4464	(0.2449,0.8139)
Natural spline of time	49	3.4308	0.307	30.9014	(16.9299,56.4029)
Natural spline of time	50	2.648	0.3177	14.1258	(7.5784,26.3296)
Natural spline of time	51	3.0508	0.3263	21.1322	(11.1479,40.0588)
Natural spline of time	52	1.9931	0.337	7.3382	(3.7908,14.2054)
Natural spline of time	53	1.587	0.3351	4.8891	(2.535,9.4291)
Natural spline of time	54	2.6923	0.2173	14.7656	(9.6445,22.6058)
Natural spline of time	55	3.3912	0.6203	29.7016	(8.8058,100.1819)
Natural spline of time	56	1.599	0.136	4.9481	(3.7903,6.4596)
Monday		0.1205	0.0273	1.1281	(1.0693,1.1901)
Tuesday		0.0149	0.0274	1.015	(0.9619,1.071)
Wednesday		0.0028	0.0274	1.0028	(0.9504,1.0581)
Thursday		-0.0784	0.0274	0.9246	(0.8762,0.9756)
Friday		-0.0354	0.0274	0.9652	(0.9148,1.0185)
Saturday		-0.0159	0.0274	0.9842	(0.9328,1.0385)
Public holiday		-0.2378	0.0235	0.7884	(0.7529,0.8255)

1. The natural spline for time has 8 degrees of freedom per year, so there are 56 estimates of the natural spline for time in the table.
2. Sunday was used as the reference during the estimation of weekday covariate.
3. Non-holiday was used as the reference during the estimation of holiday covariate.
